# Recurrent infections caused by ESBL-producing enterobacterales with or without subsequent development of distinct carbapenem-resistant phenotypes: differential driving factors and impact on outcome

**DOI:** 10.1093/jacamr/dlag054

**Published:** 2026-04-13

**Authors:** Danielle Kruse, Pamela Ny, Paul Nieberg, Mimi Lou, Annie Wong-Beringer

**Affiliations:** Department of Clinical Pharmacy, University of Southern California (USC) Alfred E. Mann School of Pharmacy and Pharmaceutical Sciences, Los Angeles, CA, USA; Department of Pharmacy, Huntington Health, Pasadena, CA, USA; Department of Pharmacy, Huntington Health, Pasadena, CA, USA; Department of Medicine—Infectious Diseases, Huntington Health, Pasadena, CA, USA; Department of Clinical Pharmacy, University of Southern California (USC) Alfred E. Mann School of Pharmacy and Pharmaceutical Sciences, Los Angeles, CA, USA; Department of Clinical Pharmacy, University of Southern California (USC) Alfred E. Mann School of Pharmacy and Pharmaceutical Sciences, Los Angeles, CA, USA; Department of Pharmacy, Huntington Health, Pasadena, CA, USA

## Abstract

**Objectives:**

The aim of this study was to identify factors contributing to distinct carbapenem-resistant phenotypes and associated healthcare utilization and outcomes in patients with ESBL-producing *Escherichia coli* (EC) or *Klebsiella pneumoniae* (KP) with or without evolved carbapenem-heteroresistance (cHR) or -resistance (CR).

**Methods:**

A retrospective longitudinal evaluation of patients with repeated isolation of clonally related EC or KP during separate encounters. Patients were grouped by carbapenem susceptibility trajectory: (1) ESBL-ESBL; (2) ESBL-cHR; and (3) ESBL-CR. Groups were compared for host and microbial characteristics, antimicrobial exposure, and outcomes.

**Results:**

30 patients (14 ESBL-ESBL, 9 ESBL-cHR, and 7 ESBL-CR) contributed 111 encounters involving 55 EC and 56 KP. Carbapenem-resistant groups demonstrated significantly greater need for feeding tubes and central venous catheters, particularly after resistance evolved from ESBL. The ESBL-CR group was primarily involved by KP from wound and respiratory sites, whereas the ESBL-ESBL and ESBL-cHR groups were predominantly EC from urine and blood. Compared to the ESBL-ESBL group, the ESBL-CR group had the most healthcare visits, whereas the ESBL-cHR group had the longest intervals between visits. Following resistance evolution, hospitalization duration increased by 3 and 1 day, respectively, for ESBL-CR and ESBL-cHR groups (*P* = 0.01), while the ESBL-cHR group experienced more ICU admissions and septic shock. The ESBL-CR group had the highest overall antimicrobial and carbapenem exposure, while the ESBL-cHR group had the lowest.

**Conclusions:**

Evolution from ESBL to carbapenem-resistant phenotypes appeared organism-specific, as EC showed patterns consistent with cHR emergence over longer intervals and lower carbapenem exposure, whereas KP more often progressed to CR from non-urinary sources with greater healthcare utilization. If confirmed in a larger cohort, these observations may inform organism-specific stewardship strategies, enhanced diagnostics for ESBL-cHR, and further investigation into non-antibiotic mechanisms driving heteroresistance.

## Introduction

Extended-spectrum beta-lactamase-producing Enterobacterales (ESBL-E) and carbapenem-resistant Enterobacterales (CRE) remain major public health threats, with United States hospitals reporting COVID-19 pandemic-associated increases of 32% in ESBL-E and 35% in CRE infections.^1^ Prior infection caused by ESBL-E is a recognized risk factor for subsequent development of infections caused by carbapenem-resistant (CR) pathogens.^2,3^ Emerging evidence suggests that evolutionary pathways to CR appear strain specific, with *Escherichia coli* (EC) more frequently developing a carbapenem-heteroresistant (cHR) phenotype characterized by subpopulation(s) within an otherwise carbapenem-susceptible strain, while *Klebsiella pneumoniae* (KP) typically acquires resistance through porin gene mutations or the uptake of carbapenemase-encoding plasmids.^4,5^ Several studies have linked the increased transmission of carbapenemase-encoding plasmids to the absence of clustered regularly interspaced short palindromic repeats (CRISPR)/CRISPR-associated protein (Cas) systems in globally recognized KP lineages (i.e. ST258) as these systems function as bacterial defence mechanisms against foreign genetic material.^6–9^ Moreover, carbapenem exposure has been shown to directly affect CRISPR-Cas system expression in KP.^10^ Regardless of the ESBL-producing organism involved, carbapenems are recommended as the treatment of choice for invasive ESBL-E infections.^11,12^ However, carbapenem use is a major driver of the emergence of CRE and may also contribute to the development of cHR.^2,13,14^

Reports of treatment failures in a subset of carbapenem-treated patients with ESBL-E infections despite the isolates appearing susceptible in standard in-vitro testing suggest a link to the presence of a cHR phenotype.^15–18^ In addition to global reports, our group previously established the local presence and clinical relevance of cHR among ESBL-E. In a community-teaching hospital, cHR was identified in 32% (55/173) of ESBL-E isolates, enriched in non-urinary sites compared to non-cHR strains.^19–22^ In our subsequent study of 25 patients with clonally related ESBL-EC isolates from two separate hospital admissions, cHR acquisition was associated with non-urinary sources, greater carbapenem exposure, and higher morbidity potential.^2^ In a genomics-focused study, we also demonstrated within-host evolution to distinct carbapenem-resistant phenotypes in ESBL-EC and ESBL-KP.^23^ However, prior studies did not integrate longitudinal clinical data across multiple healthcare encounters over time to evaluate resistance-evolution in ESBL-E species.

Therefore, our current investigation uniquely links whole genome sequencing (WGS)-confirmed within-host evolution with longitudinal clinical trajectories to (1) identify host and microbial factors, antimicrobial exposure, and timing of emergence of distinct carbapenem-resistant phenotypes from ESBL-EC or ESBL-KP in patients with repeated infection or colonization, and (2) compare healthcare utilization and clinical outcomes at recurrent episodes of organism isolation with or without evolved resistance (ESBL to ESBL versus ESBL to cHR versus ESBL to CR).

## Patients and methods

### Study design

This retrospective case-control study was conducted at a community-teaching hospital and was approved by the local institutional review board (IRB). Adult patients with a healthcare encounter between 2013 and 2024 who had growth of ESBL-E were identified from clinical microbiology records as part of our previously published WGS cohort.^23^ For the present analysis, patients were included if they met the following criteria: (1) at least one saved isolate of ESBL-EC or ESBL-KP and at least one saved isolate demonstrating ESBL, cHR, or CR phenotype during a subsequent encounter; (2) WGS-confirmed clonality between the initial (ESBL-producing) and subsequent isolate pair; and (3) availability of medical records for at least one initial and one subsequent encounter. Sample size was determined by the number of eligible patients meeting the above criteria.

### Microbiological testing

All microbiological and molecular testing, as follows, has been performed with methods and results published previously.^2,22,23^ Briefly, isolates from patients with an initial ESBL-EC or ESBL-KP and a repeat isolation of EC or KP on a subsequent healthcare encounter were first screened for clonal relatedness using rapid amplification of polymorphic DNA (RAPD)-PCR as a rapid, low-cost method to triage candidate pairs. WGS was subsequently performed using Illumina MiSeq sequencing, with genomes assembled and annotated in BV-BRC, to confirm pairs identified from RAPD-PCR by in-silico MLST and SNP-based comparisons against MLST-matched reference genomes.^23^ Downstream analyses reported elsewhere^23^ evaluated selected nonsynonymous mutations, CRISPR-Cas content,^23,24^ and plasmid-associated features, including contigs containing ESBL, carbapenemase, and conjugation genes.^23^ WGS was performed on one representative initial-subsequent pair per patient for clonality, and all eligible encounters were retained for longitudinal clinical analyses. Isolates were classified as ESBL, cHR, or CR per phenotype testing as described below, with confirmatory testing for ESBL or CR performed by the microbiology laboratory. Carbapenem heteroresistance was screened by disc diffusion and confirmed using population analysis profile (PAP) assay as previously published.^22^ Screen-positive isolates demonstrated growth within the disc diffusion inhibition zone despite meeting CLSI susceptibility breakpoints and were confirmed by reproducible subpopulation growth on carbapenem-containing Mueller-Hinton agar at 8-fold the MIC by broth microdilution.

### Study definitions

Each patient contributed at least two isolates: an initial isolate (the earliest available ESBL-EC or -KP in the biorepository) and a subsequent isolate with either continued ESBL susceptibility or evolution to cHR or CR, obtained from a separate healthcare encounter. A healthcare encounter was defined as an emergency department (ED) visit, outpatient visit, or hospital admission at the study institution (or external facility if records were accessible). Patients were stratified into three groups based on paired (initial-subsequent) phenotypes: (1) ESBL-ESBL; (2) ESBL-cHR; (3) ESBL-CR. For each encounter, only the most resistant isolate was counted. Multiple cultures from the same site were considered a single source, while distinct sites (i.e. urine and blood) were counted separately. Colonization was defined as the absence of infection-related signs or symptoms, or as documented by the treating physician in medical records. Polymicrobial infection or colonization refers to the recovery of another organism from the same site as the EC or KP isolate, whereas co-infection or co-colonization refers to the growth of an additional organism from a different site. Source change was defined as recovery of the same organism (EC or KP) from different anatomical sites between encounters, including either the addition or loss of sites. Among encounters with a source change, increased invasiveness was defined as progression to blood or respiratory sources from other sites (i.e. urine or skin) relative to the preceding encounter. Severity of sepsis was defined per Sepsis-3 definitions.^25^ Clinical response at 72 hours was assessed from the day of the first positive culture for infection episodes and categorized as partial (improvement) or complete resolution of infection-related signs or symptoms, or lack of improvement or worsening (failure). Time between encounters was measured from the discharge date of the initial encounter to the admission date of the subsequent encounter.

### Clinical data collection

Medical records were reviewed to obtain relevant demographics, laboratory data, clinical course, and outcomes for each encounter with a positive culture of ESBL-EC or -KP, irrespective of subsequent carbapenem susceptibility. A longitudinal review quantified cumulative systemic antimicrobial exposure and the number and timing of encounters from the first to last positive culture. Demographic data included age, sex, race/ethnicity, residence before admission, comorbidities, and invasive device use. Cardiovascular disease included hypertension, atrial fibrillation, congestive heart failure, and/or prior myocardial infarction. Chronic lung disease included chronic obstructive pulmonary disease, asthma, and/or interstitial lung disease. Neurologic disorders encompassed dementia, seizures, transient ischaemic attack, and/or cerebrovascular accident. Commonly used antibiotics for ESBL-E infections were categorized by class or agent, while less frequently used agents were grouped as ‘Other’. Data were managed in a Research Electronic Data Capture (REDCap) software hosted at the University of Southern California.^26^

### Data analysis

Study groups were compared across demographics and microbiological characteristics as well as antimicrobial use to determine factors contributing to the development of cHR and CR. The impact of carbapenem-resistant phenotypes on severity at presentation, healthcare utilization, and outcomes was compared across groups. Measures of healthcare utilization included the number and timing of encounters involving a positive culture, the need for intensive care unit (ICU) admission, and length of stay. Outcome measures included clinical response at 72 hours, discharge disposition relative to pre-admission residence, and in-hospital mortality. Chi-square or Fisher’s exact tests and Kruskal-Wallis tests were used to analyse categorical and continuous variables, as appropriate. Missing data were excluded from analyses and reported in table footnotes. Effect sizes and bootstrapping confidence intervals (CIs) were reported as Cramér’s V for categorical variables and Epsilon-squared (ε2) for continuous variables. With degrees of freedom equal to 2 or 3 in our study, the benchmarks of Cramér’s V suggest small effect <0.15; medium effect 0.15–0.25 and large effect above 0.25. The interpretation for ε2 is as follows: small effect ≥0.01; medium effect ≥ 0.06, and large effect ≥ 0.14. Hodges–Lehmann differences with 95% CIs were calculated for comparisons between two groups, estimating the median of the pairwise differences. Statistical analyses were performed using Prism version 10.4.2 (GraphPad Software, Boston, Massachusetts, USA) and SAS version 9.4 (SAS Institute Inc., Cary, NC, USA). A *P*-value ≤0.05 denotes statistical significance.

## Results

### Study population

**Figure 1.**
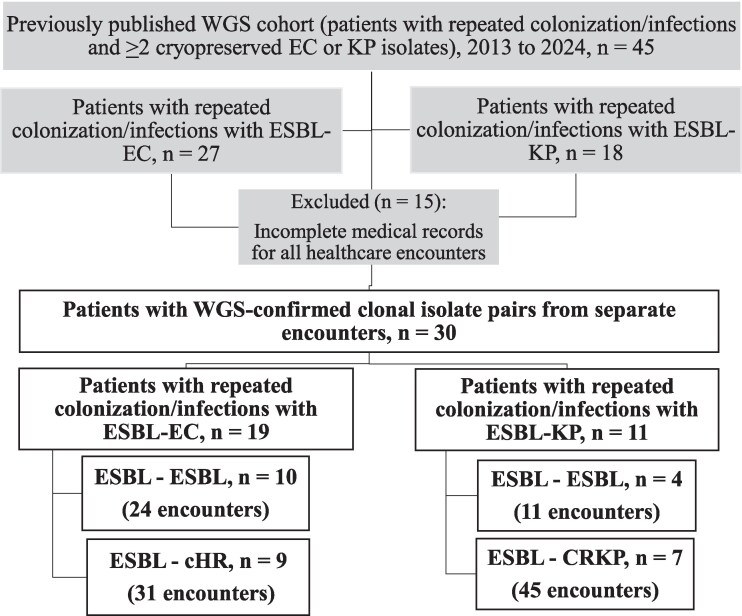
Cohort derivation from the previously published WGS dataset and selection of the encounter-based analytic cohort of ESBL-EC and -KP from patients with repeated colonization or infection, stratified by resistance phenotype. Encounter is defined as an emergency department visit, outpatient visit, or hospital admission at the study institution (or external facility if records were accessible).

Patients were selected from our previously published WGS cohort in which the clinical microbiology database was screened for healthcare encounters during the study period (2013 to 2024) with at least two cryopreserved EC or KP isolates.^23^ For the present analysis, eligible patients had at least one initial ESBL-EC or ESBL-KP isolate and at least one subsequent ESBL, cHR, or CR isolate from different encounters with WGS-confirmed clonality between the index and subsequent isolate pair and had the additional encounter-based inclusion criteria of having available medical records. A total of 30 patients were included, which comprised 14 in the ESBL-ESBL, 9 in the ESBL-cHR, and 7 in the ESBL-CR groups (Figure 1). Study patients were evaluated across 111 encounters (35 in ESBL-ESBL, 31 in ESBL-cHR, and 45 in ESBL-CR), involving 55 EC and 56 KP isolates. Previously published WGS analyses of the broader isolate set, in which the evolved isolate occurred one month to two years after the index culture (rather than being restricted to discrete encounters), confirmed clonality and characterized genetic background, CRISPR-Cas content, and evolved-strain mutations.^23^

### Risk factors for subsequent development of cHR or CR phenotypes from ESBL-EC or -KP

Clinical characteristics of the study groups at initial and/or subsequent encounters are summarized in Table 1. Compared to the ESBL-ESBL group, patients with cHR or CR phenotypes trended towards younger age (median 70–73 versus 77 years), but higher rates of diabetes (43%–56% versus 29%), chronic renal disease (44%–57% versus 29%), and neurologic disorders (43%–56% versus 29%). A trend was observed towards more ESBL-cHR and ESBL-CR patients residing in a skilled nursing facility (SNF)/long-term acute care hospital (LTACH) before encounters (27% versus 14%) and requiring an indwelling medical device (70%–71% versus 57%), such as a Foley catheter, gastrostomy/jejunostomy tube (G-tube/J-tube), central venous catheter, implantable device, or an endotracheal tube (ETT)/tracheostomy. Notably, the ESBL-cHR and ESBL-CR groups demonstrated significantly greater need for gastrostomy/jejunostomy tubes (37% and 36%) and central venous catheters (17% and 22%), compared to none reported in ESBL-ESBL (*P* = 0.002, Cramér’s V = 0.39 [95% CI, 0.26–0.40] and *P* = 0.005, Cramér’s V = 0.28 [95% CI, 0.18–0.30]). Of interest, device use increased after the development of carbapenem-resistant phenotypes, with gastrostomy/jejunostomy tubes rising from 25% (3/12) to 44% (8/18) in ESBL-cHR and from 32% (9/28) to 41% (7/17) in ESBL-CR, while central venous catheter use increased from 0% (0/12) to 28% (5/18) in ESBL cHR and from 11% (3/28) to 41% (7/17) in ESBL-CR (data not shown).

**Table 1.** Comparison of patient characteristics between study groups prior to healthcare encounters for initial and/or subsequent isolates causing infection or colonization

Characteristic	ESBL-ESBL (*n* = 14)^a^	ESBL-cHR (*n* = 9)^a^	ESBL-CR (*n* = 7)^a^	*P*-value	Effect Size (95% CI)^b^
Demographics					
Age at Encounter, years, median (IQR)	77 (62–85)	73 (62–81)	70 (64–72)	0.36	0.001 (−0.02–0.10)
BMI at Encounter^c^, kg/m^2^, median (IQR)	26 (22–29)	27 (24–33)	26 (23–27)	0.15	0.02 (−0.02–0.14)
Female, *n* (%)	9 (64)	6 (67)	4 (57)	>0.99	0.07 (0.03–0.33)
Race/Ethnicity, *n* (%)					
Non-Hispanic White	3 (21)	2 (22)	2 (29)	0.67	0.31 (0.12–0.41)
Hispanic	8 (57)	3 (33)	3 (43)		
Black or African American	3 (21)	2 (22)	2 (29)		
Asian	0 (0)	2 (22)	0 (0)		
Comorbidities, *n* (%)					
Diabetes	4 (29)	5 (56)	3 (43)	0.50	0.16 (0.04–0.38)
Cardiovascular Disease	11 (79)	7 (78)	6 (86)	>0.99	0.08 (0.03–0.33)
Chronic Renal Disease	4 (29)	4 (44)	4 (57)	0.50	0.24 (0.06–0.44)
Malignancy	6 (43)	2 (22)	3 (43)	0.62	0.20 (0.04–0.41)
Neurologic Disorder	4 (29)	5 (56)	3 (43)	0.50	0.24 (0.05–0.44)
Chronic Lung Disease	5 (36)	2 (22)	1 (14)	0.66	0.20 (0.05–0.40)
Residence Prior to Encounter^d^, *n/n* (%)					
Home^e^	26/35 (74)	18/30 (60)	31/45 (69)	0.11	0.21 (0.14–0.25)
SNF/LTACH	5/35 (14)	8/30 (27)	12/45 (27)		
B&C/ALF	2/35 (6)	4/30 (13)	0/45 (0)		
Outside Hospital	2/35 (6)	0/30 (0)	2/45 (4)		
Requires Indwelling Medical Device at Encounter^f^, *n/n* (%)	20/35 (57)	21/30 (70)	32/45 (71)	0.39	0.13 (0.02–0.24)
Foley Catheter	10/35 (29)	5/30 (17)	15/45 (33)	0.28	0.15 (0.04–0.23)
ETT/Tracheostomy	0/35 (0)	2/30 (7)	1/45 (2)	0.36	0.16 (0.09–0.24)
G-Tube/J-Tube	0/35 (0)	11/30 (37)	16/45 (36)	0.0002	0.39 (0.26–0.40)
Implantable Device	14/35 (40)	13/30 (43)	25/45 (56)	0.34	0.14 (0.04–0.24)
Central Venous Catheter	0/35 (0)	5/30 (17)	10/45 (22)	0.005	0.28 (0.18–0.30)

ALF, assisted living facility; B&C, board and care; BMI, body mass index; ETT, endotracheal tube; G-tube, gastrostomy tube; J-tube, jejunostomy tube.; LTACH, long-term acute care hospital; SNF, skilled nursing facility.

^a^Patient-level variables are reported once per patient; encounter-level variables are reported per encounter.

^b^Effect sizes: Cramér’s V for categorical variables; Epsilon-squared (ε2) for continuous variables; Hodges–Lehmann median difference (95% CI) for pairwise comparisons.

^c^BMI could not be calculated due to missing weight data for the following encounters, all involving patients in the ESBL-cHR group: one inpatient encounter at the study institution prior to electronic medical record transition (one patient), and two outpatient encounters (one patient).

^d^Residence data was missing for one outside hospital encounter (one patient) in the ESBL-cHR group.

^e^One patient in the ESBL-ESBL group was unhoused prior to both healthcare encounters.

^f^Indwelling medical device use data was missing for one inpatient encounter at the study institution prior to electronic medical record transition (one patient) in the ESBL-cHR group.

Microbial characteristics of study isolates are detailed in Table 2. The ESBL-ESBL and ESBL-cHR groups predominantly consisted of EC (69% and 100%, respectively), whereas ESBL-CR comprised only KP (100%) (*P* < 0.0001, Cramér’s V = 0.85 [95% CI, 0.53–0.89]). The site of isolation differed between groups. Urine was the most common culture site (87%, 97/111), higher in ESBL-ESBL (94%) and ESBL-cHR (97%) than ESBL-CR (76%). Other culture sources, including wound, respiratory, and blood, accounted for 18% (20/111) of study isolates, with ESBL-ESBL having the lowest proportion at 9%. Wound isolates were largely in ESBL-CR (22% versus 3% in other groups; *P* < 0.01; Cramér’s V = 0.30 [95% CI, 0.13–0.34]). Culture site changes between encounters involving urine, wound, respiratory, or blood occurred in 14% (11/81) of isolates, including 5% in ESBL-ESBL, 18% in ESBL-cHR (all but one occurred post-cHR), and 16% in ESBL-CR (half occurred post-CR) (data not shown), with urine-to-blood progression observed exclusively in ESBL-cHR and occurred post-cHR (25%). ESBL-ESBL demonstrated the highest proportion of colonization (56% versus 29% versus 29%; *P* = 0.03, Cramér’s V = 0.25 [95% CI, 0.10–0.32]) and the lowest rates of polymicrobial cultures (25% versus 29% versus 73%; *P* < 0.0001, Cramér’s V = 0.44 [95% CI, 0.25–0.46]) and co-infections or co-colonization (23% versus 28% versus 56%; *P* = 0.005, Cramér’s V = 0.31 [95% CI, 0.15–0.36]) compared with ESBL-cHR and ESBL-CR, respectively. As this study focuses on the clinical features associated with in-human evolution of resistance, readers are referred to our previously published paper^23^ for a more detailed description of the genomics findings related to lineages, CRISPR-Cas systems, and specific genetic mutations for the isolates included in this study.

**Table 2.** Comparison of microbial characteristics between study groups during healthcare encounters for initial and subsequent isolates causing infection or colonization

Characteristic	ESBL-ESBL (*n* = 35)^a^	ESBL-cHR (*n* = 31)^a^	ESBL-CR (*n* = 45)^a^	*P*-value	Effect Size (95% CI)^b^
Organism, *n/n* (%)					
*E. coli*	24/35 (69)	31/31 (100)	0/45 (0)	<0.0001	0.85 (0.53–0.89)
*K. pneumoniae*	11/35 (31)	0/31 (0)	45/45 (100)		
Source^c^, *n/n* (%)					
Urine	33/35 (94)	30/31 (97)	34/45 (76)	0.01	0.29 (0.17–0.29)
Wound	1/35 (3)	1/31 (3)	10/45 (22)	<0.01	0.30 (0.13–0.34)
Respiratory	0/35 (0)	0/31 (0)	1/45 (2)	>0.99	0.11 (0.10–0.13)
Blood	2/35 (6)	5/31 (16)	0/45 (0)	0.01	0.27 (0.12–0.33)
Source Change^d^, *n/n* (%)	1/21 (5)	4/22 (18)	6/38 (16)	0.48	0.13 (0.04–0.20)
Increased Invasiveness	0/1 (0)	1/4 (25)	0/6 (0)	0.45	0.13 (0.09–0.23)
Colonization Status^e^, *n/n* (%)	20/35 (57)	9/27 (33)	13/45 (29)	0.03	0.25 (0.10–0.32)
Polymicrobial Infection or Colonization^e^, *n/n* (%)	9/35 (26)	9/29 (31)	33/45 (73)	<0.0001	0.44 (0.25–0.46)
Co-Infection or Co-Colonization^e^, *n/n* (%)	8/35 (23)	8/29 (28)	25/45 (56)	0.005	0.31 (0.15–0.36)

^a^ *n* represents the number of healthcare encounters (one isolate per encounter) included per group; other tables report *n* as the number of patients unless otherwise specified.

^b^Effect sizes: Cramér’s V for categorical variables; Epsilon-squared (ε2) for continuous variables; Hodges–Lehmann median difference (95% CI) for pairwise comparisons.

^c^Denominator for source reflects the number of encounters per group; some encounters had multiple anatomical sources, so column totals may exceed 100%.

^d^Denominator for source change represents the total number of encounters minus one encounter per patient.

^e^Missing data on colonization, polymicrobial culture, and co-infection or co-colonization for the ESBL-cHR group: two outpatient encounters (one patient), and two inpatient encounters at the study institution prior to electronic medical record transition (one encounter each for two patients).

### Impact of carbapenem resistance development on severity at presentation, healthcare utilization, and outcome

Table 3 summarizes comparisons across groups. Patients with cHR or CR phenotypes had approximately twice as many healthcare encounters as recurrent ESBL strains (*P* ≤ 0.01, ε2 = 0.30 [95% CI, 0.05–0.67]). The interval between encounters was longest for ESBL-cHR (median 214 days) followed by ESBL-CR (median 68 days) and ESBL-ESBL (median 58 days) (*P* < 0.001, ε2 = 0.16 [95% CI, 0.03–0.33]). Encounter types differed significantly across groups (*P* = 0.01; ε2 = 0.23 [95% CI, 0.12–0.25]). Overall, most encounters required hospitalization (87%, 97/111), with the highest in ESBL-CR (98%). Twelve encounters occurred in the ED, most often in ESBL-ESBL (17%) and least in ESBL-CR (2%). Encounter length of stay differed significantly across groups (*P* < 0.01, ε2 = 0.07 [95% CI, −0.002–0.22]) and was longest for CR (median 6 days) and cHR (median 4 days), with median increases of 3 and 1 day post-evolution, respectively. Among hospitalized patients, ICU admission was more frequent in ESBL-cHR (13%) and ESBL-CR (9%) than ESBL-ESBL (3%), with all ESBL-cHR ICU admissions occurring post-evolution. Similar patterns were observed for sepsis (39% versus 40% versus 31%) and septic shock (11% versus 2% versus 3%), respectively, with all septic shock cases among ESBL-cHR and ESBL-CR occurring post-evolution. Failure to achieve stability at 72 hours occurred only in ESBL-cHR (6%). At disposition, most survivors (89%, 93/105) returned to baseline level of care. Transitions to a lower level of care were most common in ESBL-ESBL (13%), infrequent in ESBL-cHR (3%), and absent in ESBL-CR. Five in-hospital deaths occurred (3 ESBL-ESBL, 1 ESBL-cHR, 1 ESBL-CR), none attributed to infection.

**Table 3.** Outcome comparison between study groups during healthcare encounters for initial and subsequent isolates causing infection or colonization

Characteristic	ESBL-ESBL (*n* = 14)^a^	ESBL-cHR (*n* = 9)^a^	ESBL-CR (*n* = 7)^a^	*P*-value	Effect size (95% CI)^b^
Encounters, median (IQR)	2 (2–3)	4 (2–5)	5 (4–7)	<0.01	0.30 (0.05–0.67)
Time Between Encounters, days, median (IQR)	58 (39–167)	214 (160–357)	68 (38–152)	<0.001	0.16 (0.03–0.33)
Encounter Type, *n/n* (%)					
ED	6/35 (17)	5/31 (16)	1/45 (2)	0.01	0.23 (0.12–0.25)
Inpatient	29/35 (83)	24/31 (77)	44/45 (98)		
Outpatient	0/35 (0)	2/31 (6)	0/45 (0)		
Encounter Length of Stay, days, median (IQR)	4 (2–7)	4 (1–8)	6 (4–12)	<0.01	0.07 (−0.002–0.22)
Before Evolved to cHR or CR	—	3 (1–6)	5 (3–11)	0.05	2.5 (0–5)
After Evolved to cHR or CR	—	4 (1–8)	8 (5–14)	0.06	3.5 (0–7)
Severity at Presentation at Encounter^c^, *n/n* (%)					
Stable	23/35 (66)	14/28 (50)	26/45 (58)	0.44	0.14 (0.05–0.21)
Sepsis	11/35 (31)	11/28 (39)	18/45 (40)		
Septic Shock	1/35 (3)	3/28 (11)	1/45 (2)		
ICU Admission at Encounter^d^, *n/n* (%)	1/29 (3)	3/23 (13)	4/44 (9)	0.45	0.12 (0.03–0.21)
Clinical Response at 72 Hours at Encounter^e^, *n/n* (%)					
Partial	9/13 (69)	14/18 (78)	23/32 (72)	0.58	0.12 (0.02–0.17)
Complete	4/13 (31)	3/18 (17)	9/32 (28)		
Failure	0/13 (0)	1/18 (6)	0/32 (0)		
Residence at Disposition Relative to Residence Prior to Encounter^f^, *n/n* (%)					
Lower Level of Care	4/32 (13)	1/29 (3)	0/44 (0)	0.02	0.22 (0.13–0.25)
Same Level of Care	24/32 (75)	28/29 (97)	41/44 (93)		
Higher Level of Care	4/32 (13)	0/29 (0)	3/44 (7)		
In-Hospital Mortality, *n* (%)	3 (21)	1 (11)	1 (14)	>0.99	0.12 (0.03–0.34)

^a^Patient-level variables are reported once per patient; encounter-level variables are reported per encounter. Encounter is defined as an emergency department visit, outpatient visit, or hospital admission at the study institution (or external facility if records were accessible).

^b^Effect sizes: Cramér’s V for categorical variables; Epsilon-squared (ε2) for continuous variables; Hodges–Lehmann median difference (95% CI) for pairwise comparisons.

^c^Missing severity at presentation data for the ESBL-cHR group: two outpatient encounters (one patient), and one inpatient encounter at the study institution before electronic medical record transition (one patient).

^d^Missing ICU admission data for the ESBL-cHR group: one inpatient encounter at the study institution before electronic medical record transition (one patient).

^e^Missing clinical response data for the ESBL-ESBL group: two ED encounters without clinical status assessment (one patient); and the ESBL-cHR group: two outpatient encounters (one patient), and two inpatient encounters at the study institution before electronic medical record transition (one encounter each for two patients).

^f^Higher level care = admitted from home but discharged to SNF/LTACH; Same level care = admitted from home and discharged to home or admitted from SNF/LTACH and discharged to SNF/LTACH; Lower-level care = admitted from SNF/LTACH and discharged to home. Residence data were missing for one outside hospital encounter (one patient) in the ESBL-cHR group.

### Antimicrobial exposures between groups

Antimicrobial exposures across all healthcare visits, capturing antibiotic use both before and during each encounter up to the last positive culture for each group’s final phenotype (ESBL, cHR, or CR), are depicted in Table 4. Antibiotic exposure was significantly different among study groups preceding the development of carbapenem resistance, with overall exposure (3553 days) as well as carbapenem exposure (641 days) being highest in the ESBL-CR group, while fluoroquinolone exposure (864 days) was highest among ESBL-ESBL patients. Interestingly, the ESBL-cHR group had the least amount of overall antibiotic exposure (485 versus 3005 days, *P* = 0.01, data not shown) and specifically carbapenem exposure (79 versus 187 days, *P* = 0.02; ε2 = 0.23 [95% CI, −0.01–0.60]) before developing the cHR phenotype, even lower than that of the ESBL-ESBL group.

**Table 4.** Comparison of systemic antimicrobial exposure between study groups across all healthcare encounters preceding the final phenotype (ESBL, cHR, or CR) encounter

Characteristic	ESBL-ESBL (n = 14)	ESBL-cHR (n = 9)	ESBL-CR (n = 7)	*P*-value	Effect size (95% CI)^a^
		Before Evolved to cHR	Before Evolved to CR		
Carbapenems^b^, days	187	79	641	0.02	0.23 (−0.01–0.60)
Meropenem	66	48	227	0.07	0.12 (−0.06–0.51)
Ertapenem	121	31	414	≤0.01	0.12 (0.04–0.69)
Non-Carbapenem Beta-lactams, days	923	246	1102	0.15	0.07 (−0.06–0.40)
Fluoroquinolones, days	864	19	353	0.06	0.14 (−0.02–0.49)
Sulfonamides, days	114	8	198	0.37	−0.001 (−0.07–0.35)
Nitrofurantoin/Fosfomycin, days	126	12	158	0.27	0.02 (−0.06–0.37)
Others^c^, days	791	121	1101	<0.001	0.28 (0.06–0.60)

^a^Effect sizes: Epsilon-squared (ε2) for continuous variables.

^b^Carbapenems included a carbapenem/beta-lactamase inhibitor combination for one ESBL-CR patient (after evolving to CR).

^c^Other antibiotics included tetracyclines, macrolides, glycopeptides, lipoglycopeptides, metronidazole, aminoglycosides, vancomycin (oral and rectal), fidaxomicin, polymyxins, clindamycin, linezolid, and rifampin.

## Discussion

To our knowledge, this is the first study to longitudinally track clinical and microbial factors, healthcare utilization, and outcomes linked to within-host evolution of carbapenem-heteroresistant and -resistant phenotypes in ESBL-E among patients with repeated healthcare exposure. In this cohort of 30 patients with 111 encounters following initial isolation of ESBL-*E. coli* or -*K. pneumoniae*, evolution to cHR or CR appeared more common among those with greater comorbidity burden, device use, and exposure to healthcare facilities such as SNFs/LTACHs, suggesting that host vulnerability and intensive healthcare contact may facilitate emergence of these resistant phenotypes.

Anatomic source also appeared to influence evolution among ESBL-E strains. Our prior work showed that over 30% of cHR ESBL-E strains were derived from non-urinary sites (17/55),^22^ and that 27% of patients who developed cHR after recurrent ESBL-EC isolation (15/55) were more likely to have ESBL-EC recovered from both urine and blood, with the cHR-EC isolate collected from a distinct site (*P* = 0.061).^2^ Consistent with this, evolved strains in the present study were more often recovered from non-urinary sites and associated with source changes between encounters compared to those with unchanged ESBL strains, potentially reflecting enhanced adaptability and survival in diverse anatomical niches likely driven by combined genetic, phenotypic, and environmental pressures.^23^

Most EC isolates belonged to the globally dominant ST131 lineage, a high-risk clone defined by accessory genome variability and frequent partial or complete CRISPR-Cas loss, which are features implicated in the development of multidrug resistance phenotypes.^27–31^ Prior reports show ST131’s enhanced ability to colonize or invade non-urinary tissues and cause severe infections.^32–36^ In our cohort, CRISPR-Cas loss was common, but did not distinguish isolates that remained ESBL-only from those that evolved. However, cHR isolates exhibited enrichment of metabolic and virulence-associated modifications, and, to a lesser extent, outer-membrane adaptations, features less common among ESBL-only isolates.^23^ Such adaptations enhance persistence and survival outside the bladder and likely contributed to the observed source shifts and increased recovery from non-urinary and invasive sites.^37–40^

In contrast, KP evolution appeared lineage-associated, with ST258 absent among ESBL-ESBL but present among ESBL-CRKP pairs, consistent with reports linking ST258 to CRKP, hospital-associated infections (particularly pneumonia), and skin and soft tissue infections.^9,41,42^ ST258 strains commonly exhibit CRISPR-Cas loss and high plasmid permissiveness, facilitating acquisition and stable maintenance of carbapenemase-encoding multidrug resistance plasmids.^9,43^ Among our KP isolates, carbapenemase acquisition occurred only in evolved ESBL-CRKP isolates and coincided with acquisition of an IncFIB plasmid, often without additional mutations as reported previously,^23^ suggesting carbapenemase alone may confer resistance. For carbapenemase-negative CRKP, porin disruptions predominated, consistent with reduced carbapenem entry as a key mechanism.^44^ ESBL-KP isolates that remained ESBL lacked carbapenemase acquisition and porin mutations and showed limited additional genomic changes, consistent with relative stability.

Interestingly, cHR-EC isolates emerged under lower prior carbapenem exposure and were associated with longer intervals between episodes compared with ESBL-EC isolates that remained ESBL. This supports a persistence-oriented adaptive pathway in which altered outer membrane permeability and metabolic rewiring enable survival under low or intermittent antibiotic exposure by shifting into a slow-growth, stress-tolerant state. Such tolerance can precede and facilitate resistance evolution, consistent with a persistence-driven rather than antibiotic exposure-driven evolution.^45–47^ Experimental studies have similarly shown that low or fluctuating concentrations of imipenem or ertapenem can induce heteroresistant EC subpopulations.^48,49^ In contrast, our previous work observed higher doses (*P* = 0.38) and longer durations (*P* = 0.51) of carbapenem therapy prior to cHR evolution.^2^ This discrepancy likely reflects methodological differences, as the prior analysis examined only the treatment interval most proximal to cHR isolation, whereas the present longitudinal design captured carbapenem use across pre-ESBL, all ESBL episodes, and inter-episode intervals, thereby distributing exposure more broadly, particularly among patients with multiple ESBL episodes. Conversely, ESBL-CRKP patients had the highest carbapenem exposure prior to CR development, consistent with the well-recognized role of sustained carbapenem pressure in selecting for full resistance. Numerous prospective and retrospective studies identified prior carbapenem use as an independent risk factor for CRKP isolation.^50–52^ Collectively, these findings suggest distinct adaptive trajectories between EC and KP, as EC may evolve towards cHR through chromosomal host-adaptive remodelling under lower selective pressure, whereas KP may progress to CR either through carbapenemase acquisition or porin disruption under sustained carbapenem exposure. These patterns underscore the need for enhanced diagnostics to detect heteroresistance and for antimicrobial stewardship interventions to prioritize carbapenem-sparing strategies where appropriate.

Regarding clinical outcomes, the cHR group appeared more likely to present with sepsis or septic shock, largely post-cHR, require ICU admission (exclusively post-cHR), and exhibit the lowest proportion of complete clinical response at 72 hours. CR patients had the highest healthcare contact intensity, with the greatest number of encounters, nearly universal hospitalization, and prolonged lengths of stay, which further increased after CR emergence. These trends raise the possibility that within-host evolution to cHR or CR may contribute to increased healthcare resource consumption and poorer short-term outcomes, highlighting the need for identifying high-risk populations and implementing proactive antimicrobial stewardship interventions aimed at reducing morbidity and improving outcomes, such as flagging patients with prior ESBL/CR infections, monitoring those with high cumulative carbapenem exposure, promoting carbapenem de-escalation protocols, and testing for cHR.

This study has several limitations. Its retrospective design may have incompletely captured antibiotic exposures (particularly outside admissions or outpatient prescribing), healthcare utilization outside our health system, and variability in culture sampling. Nonetheless, it is the first to comprehensively capture drug exposure metadata across encounters to evaluate cumulative antibiotic contribution to cHR and CR. The small sample size and imbalance in EC and KP isolates per group limit interpretation but reflect the real-world frequency of these resistant phenotypes. Similarly, the limited number of isolates evaluated in this study may amplify strain- and patient-specific variability, thus findings should be viewed as descriptive and warrant confirmation in larger cohorts. Multivariable modelling was not feasible, and associations between specimen source and resistance evolution may be confounded by sampling frequency and infection-specific antibiotic exposure. Therefore, comparisons across groups were unadjusted for baseline differences and observed associations should be interpreted as exploratory. However, restricting inclusion to patients with repeated encounters involving clonally related isolates allowed direct linkage of clinical drivers to within-host ESBL-E evolution, providing a robust framework to longitudinally study the clinical impact of carbapenem-resistant phenotypes on patient and microbial outcomes. Future studies with larger cohorts may better distinguish the contribution of individual antibiotics within the same class, particularly meropenem and ertapenem, to this evolution and delineate whether distinct agents impose differential evolutionary pressures on ESBL-E strains.

In summary, patients with repeated ESBL-EC or ESBL-KP isolation, especially from non-urinary sites and with prior carbapenem exposure, appear at increased risk for subsequent cHR or CR over time. The results of the present investigation warrant further confirmation with a larger sample size but highlight the potential risk for increased morbidity associated with evolved resistance. They emphasize the importance of close monitoring and consideration of non-carbapenem treatment options for recurrent ESBL-EC or ESBL-KP infections, especially in noncritically ill patients, to limit the subsequent selection of carbapenem-resistant phenotypes. Treatment strategies tailored by strain-specifics may be warranted, such as targeted testing for cHR and early transition to a non-carbapenem agent in recurrent ESBL-EC infections with inadequate clinical response to carbapenem therapy, given the distinct evolutionary pathways observed between *E. coli* and *K. pneumoniae* lineages.

## References

[1] CDC . COVID-19: U.S. Impact on Antimicrobial Resistance, Special Report 2022. U.S. Department of Health and Human Services, CDC, 2022. https://www.cdc.gov/antimicrobial-resistance/media/pdfs/covid19-impact-report-508.pdf.

[2] Tan K, Kelsom C, Chron A et al Risk factors and outcome associated with infection or colonization due to carbapenem-heteroresistant *Escherichia coli*. JAC Antimicrob Resist 2021; 3: 1–7. 10.1093/jacamr/dlab036 PMC8210097 34223107

[3] Ny P, Nieberg P, Wong-Beringer A. Impact of carbapenem resistance on epidemiology and outcomes of nonbacteremic *Klebsiella pneumoniae* infections. Am J Infect Control 2015; 43: 1076–80. 10.1016/j.ajic.2015.06.008 26190386

[4] Logan LK, Weinstein RA. The epidemiology of carbapenem-resistant Enterobacteriaceae: the impact and evolution of a global menace. J Infect Dis 2017; 215: S28–36. 10.1093/infdis/jiw282 28375512 PMC5853342

[5] Sun JD, Huang SF, Yang SS et al Impact of carbapenem heteroresistance among clinical isolates of invasive *Escherichia coli* in Chongqing, southwestern China. Clin Microbiol Infect 2015; 21: 469.e1–469.e10. 10.1016/j.cmi.2014.12.013 25649300

[6] Westra ER, Swarts DC, Staals RHJ et al The CRISPRs, they are A-Changin’: how prokaryotes generate adaptive immunity. Annu Rev Genet 2012; 46: 311–39. 10.1146/annurev-genet-110711-155447 23145983

[7] Li HY, Kao CH, Lin WH et al Characterization of CRISPR-Cas systems in clinical *Klebsiella pneumoniae* isolates uncovers its potential association with antibiotic susceptibility. Front Microbiol 2018; 9: 1–9. 10.3389/fmicb.2018.01595 30061876 PMC6054925

[8] Wyres KL, Wick RR, Judd LM et al Distinct evolutionary dynamics of horizontal gene transfer in drug resistant and virulent clones of *Klebsiella pneumoniae*. PLOS Genet 2019; 15: 1–25. 10.1371/journal.pgen.1008114 PMC6483277 30986243

[9] Mackow NA, Shen J, Adnan M et al CRISPR-Cas influences the acquisition of antibiotic resistance in *Klebsiella pneumoniae*. PLoS One 2019; 14: 1–13. 10.1371/journal.pone.0225131 PMC6867608 31747398

[10] Lin TL, Pan YJ, Hsieh PF et al Imipenem represses CRISPR-Cas interference of DNA acquisition through H-NS stimulation in *Klebsiella pneumoniae*. Sci Rep 2016; 6: 1–10. 10.1038/srep31644 27531594 PMC4987720

[11] Tamma PD, Heil EL, Justo JA et al Infectious Diseases Society of America 2024 guidance on the treatment of antimicrobial-resistant gram-negative infections. Clin Infect Dis 2024: 1–56. 10.1093/cid/ciae403 39108079

[12] Karaiskos I, Giamarellou H. Carbapenem-sparing strategies for ESBL producers: when and how. Antibiotics 2020; 9: 1–23. 10.3390/antibiotics9020061 PMC7167803 32033322

[13] Adams-Sapper S, Nolen S, Donzelli GF et al Rapid induction of high-level carbapenem resistance in heteroresistant KPC-producing *Klebsiella pneumoniae*. Antimicrob Agents Chemother 2015; 59: 3281–9. 10.1128/AAC.05100-14 25801565 PMC4432212

[14] Nicoloff H, Hjort K, Levin BR et al The high prevalence of antibiotic heteroresistance in pathogenic bacteria is mainly caused by gene amplification. Nat Microbiol 2019; 4: 504–14. 10.1038/s41564-018-0342-0 30742072

[15] Harris PNA, Tambyah PA, Lye DC et al Effect of piperacillin-tazobactam vs meropenem on 30-day mortality for patients with *E. coli* or *Klebsiella pneumoniae* bloodstream infection and ceftriaxone resistance: a randomized clinical trial. JAMA 2018; 320: 984–94. 10.1001/jama.2018.12163 30208454 PMC6143100

[16] Pilmis B, Parize P, Zahar JR et al Alternatives to carbapenems for infections caused by ESBL-producing Enterobacteriaceae. Eur J Clin Microbiol Infect Dis 2014; 33: 1263–5. 10.1007/s10096-014-2094-y 24691683

[17] Seo YB, Lee J, Kim YK et al Randomized controlled trial of piperacillin-tazobactam, cefepime and ertapenem for the treatment of urinary tract infection caused by extended spectrum β-lactamase-producing *Escherichia coli*. BMC Infect Dis 2017; 17: 1–9. 10.1186/s12879-017-2502-x 28592240 PMC5463388

[18] Band VI, Weiss DS. Heteroresistance: a cause of unexplained antibiotic treatment failure? PLoS Pathog 2019; 15: 1–7. 10.1371/journal.ppat.1007726 PMC6553791 31170271

[19] Nodari CS, Ribeiro VB, Barth AL. Imipenem heteroresistance: high prevalence among Enterobacteriaceae *Klebsiella pneumoniae* carbapenemase producers. J Med Microbiol 2015; 64: 124–6. 10.1099/jmm.0.081869-0 25351714

[20] Pournaras S, Kristo I, Vrioni G et al Characteristics of meropenem heteroresistance in *Klebsiella pneumoniae* carbapenemase (KPC)-producing clinical isolates of *K. pneumoniae*. J Clin Microbiol 2010; 48: 2601–4. 10.1128/JCM.02134-09 20504985 PMC2897536

[21] Tato M, Morosini M, García L et al Carbapenem heteroresistance in VIM-1-producing *Klebsiella pneumoniae* isolates belonging to the same clone: consequences for routine susceptibility testing. J Clin Microbiol 2010; 48: 4089–93. 10.1128/JCM.01130-10 20844213 PMC3020823

[22] Tan K, Nguyen J, Nguyen K et al Prevalence of the carbapenem-heteroresistant phenotype among ESBL-producing *Escherichia coli* and *Klebsiella pneumoniae* clinical isolates. J Antimicrob Chemother 2020; 75: 1506–12. 10.1093/jac/dkaa048 32181802

[23] Kalu MC, Acharya A, Jorth P et al ESBL-Producing *Escherichia coli* and *Klebsiella pneumoniae* exhibit divergent paths during in-human evolution towards carbapenem resistance. Microorganisms 2025; 13: 1–18. 10.3390/microorganisms13061387 PMC12196164 40572275

[24] Couvin D, Bernheim A, Toffano-Nioche C et al CRISPRCasFinder, an update of CRISRFinder, includes a portable version, enhanced performance and integrates search for Cas proteins. Nucleic Acids Res 2018; 46: W246–51. 10.1093/nar/gky425 29790974 PMC6030898

[25] Singer M, Deutschman CS, Seymour CW et al The third international consensus definitions for sepsis and septic shock (sepsis-3). JAMA 2016; 315: 801–10. 10.1001/jama.2016.0287 26903338 PMC4968574

[26] Harris PA, Taylor R, Thielke R et al Research electronic data capture (REDCap): a metadata-driven methodology and workflow process for providing translational research informatics support. J Biomed Inform 2009; 42: 377–81. 10.1016/j.jbi.2008.08.010 18929686 PMC2700030

[27] Lee D, Muir P, Lundberg S et al A CRISPR-Cas9 system protecting *E. coli* against acquisition of antibiotic resistance genes. Sci Rep 2025; 15: 1–10. 10.1038/s41598-025-85334-2 39789078 PMC11718013

[28] Decano AG, Tran N, Al-Foori H et al Plasmids shape the diverse accessory resistomes of *Escherichia coli* ST131. Access Microbiol 2020; 3: 1–15. 10.1099/acmi.0.000179 PMC8115979 33997610

[29] Aydin S, Personne Y, Newire E et al Presence of type I-F CRISPR/Cas systems is associated with antimicrobial susceptibility in *Escherichia coli*. J Antimicrob Chemother 2017; 72: 2213–8. 10.1093/jac/dkx137 28535195

[30] Favate JS, Skalenko KS, Chiles E et al Linking genotypic and phenotypic changes in the *E. coli* long-term evolution experiment using metabolomics. eLife 2023; 12: 1–15. 10.7554/eLife.87039 PMC10665018 37991493

[31] Lopatkin AJ, Bening SC, Manson AL et al Clinically relevant mutations in core metabolic genes confer antibiotic resistance. Science 2021; 371: 1–22. 10.1126/science.aba0862 PMC8285040 33602825

[32] Brumwell A, Sutton G, Lantos PM et al *Escherichia coli* ST131 associated with increased mortality in bloodstream infections from urinary tract source. J Clin Microbiol 2023; 61: 1–9. 10.1128/jcm.00199-23 PMC10358158 37338371

[33] Drawz SM, Porter S, Kuskowski MA et al Variation in resistance traits, phylogenetic backgrounds, and virulence genotypes among *Escherichia coli* clinical isolates from adjacent hospital campuses serving distinct patient populations. Antimicrob Agents Chemother 2015; 59: 5331–9. 10.1128/AAC.00048-15 26100703 PMC4538515

[34] Price LB, Johnson JR, Aziz M et al The epidemic of extended-spectrum-β-lactamase producing *Escherichia coli* ST131 is driven by a single highly pathogenic subclone, H30-Rx. mBio 2013; 4: 1–10. 10.1128/mBio.00377-13 PMC3870262 24345742

[35] Yao ZC, Sun Y, Ma ZX et al Longitudinal analysis of carbapenem-resistant *Escherichia coli* in a Southern Chinese hospital (2015–2021): epidemiology, genetics, and resistance mechanisms. Int J Antimicrob Agents 2025; 66: 1–9. 10.1016/j.ijantimicag.2025.107577 40692074

[36] Dagher C, Salloum T, Alousi S et al Molecular characterization of Carbapenem resistant *Escherichia coli* recovered from a tertiary hospital in Lebanon. PLoS One 2018; 13: 1–13. 10.1371/journal.pone.0203323 PMC6126819 30188911

[37] Johnson JR, Clabots C, Porter SB et al Intestinal persistence of colonizing *Escherichia coli* strains, especially ST131-H30, in relation to bacterial and host factors. J Infect Dis 2022; 225: 2197–207. 10.1093/infdis/jiab638 34979558 PMC9200155

[38] Totsika M, Beatson SA, Sarkar S et al Insights into a multidrug resistant *Escherichia coli* pathogen of the globally disseminated ST131 lineage: genome analysis and virulence mechanisms. PLoS One 2011; 6: 1–11. 10.1371/journal.pone.0026578 PMC3203889 22053197

[39] Schäfer F, Görner P, Woltemate S et al The resistance mechanism governs physiological adaptation of *Escherichia coli* to growth with sublethal concentrations of Carbapenem. Front Microbiol 2022; 12: 1–10. 10.3389/fmicb.2021.812544 PMC8841762 35173695

[40] Heffernan JR, Wildenthal JA, Tran H et al Yersiniabactin is a quorum-sensing autoinducer and siderophore in uropathogenic *Escherichia coli*. mBio 2024; 15: 1–17. 10.1128/mbio.00277-23 PMC10865836 38236035

[41] Wong Fok Lung T, Charytonowicz D, Beaumont KG et al *Klebsiella pneumoniae* induces host metabolic stress that promotes tolerance to pulmonary infection. Cell Metab 2022; 34: 761–74.e9. 10.1016/j.cmet.2022.03.009 35413274 PMC9081115

[42] Dhar S, Martin ET, Lephart PR et al Risk factors and outcomes for carbapenem resistant *Klebsiella pneumoniae* isolation, stratified by its multilocus sequence typing: ST258 versus non-ST258. Open Forum Infect Dis 2016; 3: 1–5. 10.1093/ofid/ofv213 PMC4751918 26885543

[43] Chen L, Mathema B, Pitout JDD et al Epidemic *Klebsiella pneumoniae* ST258 is a hybrid strain. mBio 2014; 5: 1–8. 10.1128/mBio.01355-14 PMC4073492 24961694

[44] Mancuso G, De Gaetano S, Midiri A et al The challenge of overcoming antibiotic resistance in carbapenem-resistant gram-negative Bacteria: “attack on titan”. Microorganisms 2023; 11: 1–16. 10.3390/microorganisms11081912 PMC10456941 37630472

[45] Levin-Reisman I, Ronin I, Gefen O et al Antibiotic tolerance facilitates the evolution of resistance. Science 2017; 355: 826–30. 10.1126/science.aaj2191 28183996

[46] Levin-Reisman I, Brauner A, Ronin I et al Epistasis between antibiotic tolerance, persistence, and resistance mutations. Proc Natl Acad Sci U S A 2019; 116: 14734–9. 10.1073/pnas.1906169116 31262806 PMC6642377

[47] Hussein K, Sprecher H, Mashiach T et al Carbapenem resistance among *Klebsiella pneumoniae* isolates: risk factors, molecular characteristics, and susceptibility patterns. Infect Control Hosp Epidemiol 2009; 30: 666–71. 10.1086/598244 19496647

[48] Tängdén T, Adler M, Cars O et al Frequent emergence of porin-deficient subpopulations with reduced carbapenem susceptibility in ESBL-producing *Escherichia coli* during exposure to ertapenem in an in vitro pharmacokinetic model. J Antimicrob Chemother 2013; 68: 1319–26. 10.1093/jac/dkt044 23478794

[49] Luo Y, Xu R, Yuan B et al Heterogeneous subpopulations in *Escherichia coli* strains acquire adaptive resistance to imipenem treatment through rapid transcriptional regulation. Front Cell Infect Microbiol 2025; 15: 1–13. 10.3389/fcimb.2025.1563316 PMC12163615 40521030

[50] Patel G, Huprikar S, Factor SH et al Outcomes of carbapenem-resistant *Klebsiella pneumoniae* infection and the impact of antimicrobial and adjunctive therapies. Infect Control Hosp Epidemiol 2008; 29: 1099–106. 10.1086/592412 18973455

[51] Kwak YG, Choi SH, Choo EJ et al Risk factors for the acquisition of carbapenem resistant *Klebsiella pneumoniae* among hospitalized patients. Microb Drug Resist 2005; 11: 165–9. 10.1089/mdr.2005.11.165 15910232

[52] Hu Y, Ping Y, Li L et al A retrospective study of risk factors for carbapenem-resistant *Klebsiella pneumoniae* acquisition among ICU patients. J Infect Dev Ctries 2016; 10: 208–13. 10.3855/jidc.6697 27031451

